# Consecutive versus separate sessions of endoscopic ultrasound (EUS) and endoscopic retrograde cholangiopancreatography (ERCP) for symptomatic choledocholithiasis

**DOI:** 10.1007/s00464-012-2720-7

**Published:** 2013-02-07

**Authors:** Fabiana Benjaminov, Assaf Stein, George Lichtman, Itamar Pomeranz, Fred M. Konikoff

**Affiliations:** Gastroenterology and Hepatology Department, Meir Medical Center, Tchernichovsky 59, 77456 Kfar-Saba, Israel

**Keywords:** EUS, ERCP, CBD, Choledocholithiasis

## Abstract

**Background:**

Common bile duct (CBD) stones are a potentially life-threatening medical condition. Patients with proven CBD stones should undergo stone extraction. The aim of this study was to evaluate whether performing endoscopic ultrasound (EUS) and endoscopic retrograde cholangiopancreatography (ERCP) for symptomatic CBD stones in a single session reduces complications related to postponing treatment due to separate EUS and ERCP sessions, and to assess the safety in both options.

**Methods:**

A total of 151 patients with EUS-proven CBD stones, with subsequent ERCP, treated in our department between January 2005 and December 2011 were included. Complications related to the procedures or sedation and complications due to the CBD stones when EUS and ERCP were not performed in a single session were assessed and compared to complications when the two procedures were performed in one session.

**Results:**

In total, 149 patients of the 151 (98.7 %) had a successful ERCP. Four (5 %) patients in the separate-session group (B) had a major complication compared to none in the single-session group (A) (*p* > 0.05). Group B received 14 % more midazolam during ERCP than group A (*p* < 0.05). No sedation-related complications were noted in either group. Eleven of the 80 patients in group B (13.8 %) experienced complications while waiting for ERCP compared to none in group A (*p* = 0.001, OR = 2.17, CI = 1.06–4.

**Conclusions:**

EUS and ERCP done in a single session proved to be safe, with no increase in sedation- or procedure-related complications. Postponing treatment for symptomatic CBD stones exposes the patient to biliary complications, especially cholangitis.

The prevalence of cholelithiasis in Western countries ranges between 10 and 20 % [1]. Among these patients, common bile duct (CBD) stones are present in up to 15–20 % [2]. The natural history of CBD stones is not well known, but they may lead to serious complications such as severe abdominal pain, biliary pancreatitis, obstructive jaundice, ascending cholangitis, and hepatic abscess formation [3]. Therefore, patients with proven CBD stones should undergo stone extraction, either by endoscopic retrograde cholangiopancreatography (ERCP) or by intraoperative bile duct examination during cholecystectomy [3]. In order not to expose patients to unnecessary invasive interventions, it is recommended that patients with a moderate to high index of suspicion for CBD stones undergo prior noninvasive evaluation of their CBD by endoscopic ultrasound (EUS) or magnetic resonance imaging (MRI) [4].

Performing EUS before ERCP can prevent two thirds of unnecessary ERCPs [5]. A recent study showed that ERCP for CBD stone extraction after a positive EUS for low- to moderate-risk patients, performed during the same endoscopic session, is safe and efficacious compared to when the sessions are performed separately [6].

The aim of our study was to evaluate whether performing EUS and ERCP for symptomatic CBD stones in a single session will reduce complications related to postponing treatment due to separate EUS and ERCP sessions, and to assess the safety in both options.

## Patients and methods

All EUS studies performed in our department from January 2005 to December 2011 were reviewed. Those reporting CBD stones and followed by an ERCP were included and reviewed. The following data were recorded: demographics, comorbidities, relevant medication history, time elapsed between the EUS and ERCP (if separate sessions were performed), complications related to the procedures or sedation, complications due to the CBD stones when EUS and ERCP were not performed in a single session, gallbladder status, and the presence of a periampullary diverticulum.

The EUS studies were performed with a Pentax linear array echoendoscope (EG-3870UTK or EG-3830UTK), and the ERCP studies were performed with Olympus TJF 160VR duodenoscopes. Patients who had consecutive sessions of EUS and ERCP were transferred from the EUS suite to the ERCP suite in the same endoscopy unit. All procedures were done by experienced interventional endoscopists.

### Statistical analysis

Categorical data are presented as numbers and percentage and continuous data as mean ± standard deviation. Differences between nominal data were compared by χ^2^. Risk factors were calculated by the OR (odds ratio) and 95 % CI (confidence interval). Continuous data were compared using the *t* test. A *p* value <0.05 was considered statistically significant. All statistical analyses were performed using SPSS v19 software (SPSS, Inc., Chicago, IL).

## Results

**Fig. 1:**
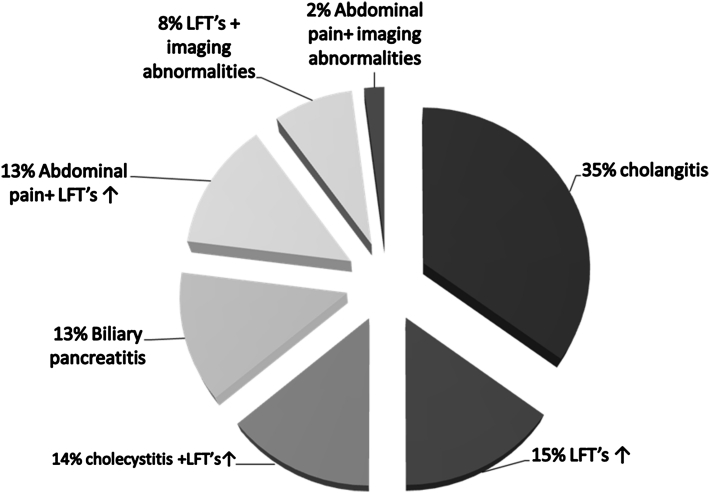
Indications for EUS

A total of 151 patients with CBD stones confirmed by EUS where included in the study. The patients were divided into two groups (Table 1). Group A (71 patients, 43.7 % men) underwent the EUS and ERCP in a single session and group B (80 patients, 52.5 % men) had the two procedures in separate sessions (*p* > 0.05), with a median time from EUS to ERCP of 7 days (range = 2–97). The mean age in groups A and B was 58.2 ± 18.4 and 67.7 ± 15.7 years, respectively (*p* = 0.001). No difference was noted between the two groups regarding diabetes, hypertension, dyslipidemia, use of aspirin or cholesterol-lowering drugs, gallbladder status, presence of a periampullary diverticulum, and CBD diameter (Table 1). Indications for EUS in all patients are detailed in Fig. 1, with no differences found between groups A and B.

**Table 1:** Group characteristics

	Group A (*n* = 71)	Group B (*n* = 80)	*p* value
Men	31 (43.7 %)	42 (52.5 %)	NS
Age (years)	58.2 ± 18.4	67.7 ± 15.7	<0.01
CBD (mm)	7.5 ± 2.5	7.7 ± 2.4	NS
Post cholecystectomy	15 (21.1 %)	22 (27.5 %)	NS
Periampullary diverticulum	15 (21.1 %)	22 (27.5 %)	NS
Aspirin use	18 (25 %)	32 (40 %)	NS
Cholesterol-lowering drugs	22 (31 %)	34 (42 %)	NS

ERCP characteristics are detailed in Table 2. In total, 149 patients of the 151 (98.7 %) had a successful ERCP. Stones were removed from 126 of the 151 patients (83.4 %): 88.7 % of group A and 78.8 % of group B (*p* > 0.05). All patients had a sphincterotomy performed, and three patients needed the assistance of a precut procedure (1 in group A and 2 in group B). Seventy of the 71 patients (98.6 %) in group A and 73 of the 80 (91.2 %) in group B had their stones removed with an extraction balloon. One patient (1.4 %) in group A and 7 (8.8 %) in group B had their stones extracted with a basket (*p* > 0.05). One patient in each group had a failed ERCP and the stones had to be removed in surgery. Two patients in group B needed a rendezvous procedure to complete the ERCP and stone extraction. Three patients in group B needed more than one ERCP for the complete extraction of all CBD stones. Minor complications were present in 11 (15.7 %) and 13 (16.2 %) patients in groups A and B, respectively (*p* > 0.05) and included postsphincterotomy bleeding that stopped spontaneously or with balloon tamponade or mild pancreatitis with spontaneous resolution. Four (5 %) patients in group B had a major complication. One had a severe postsphincterotomy hemorrhage necessitating admission, blood transfusions, and endoscopic intervention. One patient had a perforation treated by surgery and made a full recovery. Two patients died from severe post-ERCP pancreatitis. No major complications were noted in group A (*p* > 0.05).

**Table 2:** ERCP characteristics

Group	A (*n* = 71)	B (*n* = 80)	*p* value
Successful ERCP	70 (98.5 %)	79 (98.7 %)	NS
>1 ERCP	0	3 (3.7 %)	NS
Stone removal	63 (88.7 %)	63 (78.8 %)	NS
Minor complications	11 (15.7 %)	13 (16.2 %)	NS
Major complications	0	4 (5 %)	NS

All patients were sedated with midazolam and fentanyl. During EUS, patients in group A received 4.6 ± 1.4 mg midazolam and 84 ± 19 μg fentanyl compared to patients in group B who received 4.4 ± 1.4 mg midazolam and 81 ± 21 μg fentanyl (*p* = NS for both). During ERCP, patients in group A received 5.7 ± 2 mg midazolam and 92 ± 24 μg fentanyl and patients in group B received 6.5 ± 2.1 mg midazolam and 97 ± 21 μg fentanyl (*p* < 0.02 for midazolam and *p* = NS for fentanyl). A 14 % increase in the midazolam dose for ERCP was recorded in group B. No sedation-related complications were noted in either group.

Complications that occurred between EUS and ERCP are shown in Table 3. Eleven of the 80 patients in group B (13.8 %) experienced complications while waiting for ERCP compared to none in group A (*p* = 0.001, OR = 2.17, CI = 1.06–4.4). Six patients experienced ascending cholangitis, four had biliary colic and elevated liver enzymes, and one had pancreatitis. Approximately 60 % of the complications occurred within 30 days of EUS. All 11 patients were treated successfully with ERCP, sphincterotomy, and stone extraction.

**Table 3:** Complications during waiting period

Group	A (*n* = 71)	B (*n* = 80)
Total	0	11 (14 %)*
Ascending cholangitis	−	6/11 (55 %)
Biliary colic + LFTs↑	−	4/11 (36 %)
Biliary pancreatitis	−	1/11 (9 %)

*OR* 2.17, *CI* 1.06–4.4

* *p* = 0.001

## Discussion

In the present study we have demonstrated that performing consecutive EUS and ERCP for symptomatic CBD stones in a single session is feasible and safe, with no increase in procedure- or sedation-related complications. Moreover, our data reveal an increased risk of performing ERCP in a separate session causing postponing treatment of these patients. Delaying ERCP resulted in significant biliary complications (including cholangitis and pancreatitis) in 14 % of patients.

CBD stones are a potentially life-threatening medical condition. Prompt treatment is recommended and urgency varies depending on the severity of the manifestation of CBD stones [7]. In the last decade EUS has become the procedure of choice for detecting CBD stones [8]. Its use prevents about 60 % of unnecessary ERCPs in patients with suspected CBD stones [5]. ERCP is reserved only for therapeutic procedures [5] because of its invasive nature and accompanying complications, especially when a sphincterotomy is performed [9].

Previous studies have demonstrated the safety of performing EUS and ERCP in a single session. These studies included patients with a variety of pancreaticobiliary conditions such as malignant tumors (pancreas, bile ducts, and ampulla), benign biliary strictures, pancreatic cysts, and chronic pancreatitis [10]. They evaluated the safety and cost-effectiveness in terms of procedural and anesthetic complications and found that performing the two procedures in one session is safe. Ross et al. [12] evaluated 114 patients who underwent EUS and ERCP in a single session but they did not compare it with performing the procedures in separate sessions. Moreover, 70 % of their patients had a malignancy while only 1.7 % had CBD stones. Studies [10, 11, 13] that have compared single versus separate sessions of EUS and ERCP were based on small samples (35–85 patients), with a minority of patients having CBD stones. None of these studies evaluated the consequence of morbidity due to postponing the treatment for symptomatic CBD. Our study included 151 patients in two groups, one had single session and the other had separate sessions, and all of whom had CBD stones. We demonstrated an OR of 2.17 for developing CBD stone-related complications when postponing treatment with a separate ERCP session. The ERCP success rate was similar in both groups (98.5 vs. 98.7 %) with similar success in stone extraction (group A, 88.7 %; group B, 78.8 %, *p* > 0.05). Fewer stones were extracted from the CBD in the separate-session group, although the difference was not statistically significant. This may mean that there was spontaneous stone passage. The sensitivity of cholangiography for CBD stones during ERCP is imperfect, ranging between 89 and 93 % [14]. False-negative ERCP usually occurs when small stones are present in a dilated duct [15]. The mean waiting period between EUS and ERCP in our study was 7 days (range = 2–97), similar to that reported by Aslanian et al. [13]. Group B was older than group A (58.2 ± 18.4 vs. 67.7 ± 15.7 years, *p* < 0.05); revising the charts showed no consideration bias except for convenience of the admitting and gastroenterology departments. No difference was noted in our study between groups A and B in procedural complication rates, which were similar to the rates reported in other studies [5, 12]. As in other studies, no sedation-related complications were noted. The overall sedation doses of midazolam and fentanyl were similar to those given in other studies [10, 13]. Patients in group B received more midazolam during ERCP than patients in group A (6.5 ± 2.1 vs. 5.7 ± 2 mg, *p* < 0.05); the patients in group A were still partly sedated from the EUS performed before ERCP.

One might argue that we could have used more MRCP and less EUS for the detection of CBD stones. As mentioned before, EUS and MRCP are considered equal in terms of CBD stone detection [4]. In our study, we chose to investigate patients who underwent EUS and not MRCP for the following reasons: (1) in our country EUS is more available and less costly than MRCP ($375 compared to $551, respectively). (2) As described previously in several studies, EUS is more accurate than MRCP for the detection of small CBD stones [16–18]. (3) Our aim was to provide a comprehensive one-step approach for patients with suspected CBD stones. Performing EUS for the detection of such stones allows us to continue directly with treatment during the same session.

In conclusion, to our knowledge, our study is the first one to compare a large number of patients with an exclusive diagnosis of CBD stones who underwent single versus separate sessions of EUS and ERCP. We have demonstrated that it is safe to perform EUS and ERCP in a single session with no increase in sedation- or procedure-related complications. Postponing treatment for symptomatic CBD stones exposes the patient to biliary complications, especially cholangitis. Our data support the notion of establishing an integrated gastroenterology unit that can manage CBD stones by a combined approach.
